# Modification of the Tumor Microenvironment Enhances Anti-PD-1 Immunotherapy in Metastatic Melanoma

**DOI:** 10.3390/pharmaceutics14112429

**Published:** 2022-11-10

**Authors:** Guilan Shi, Megan Scott, Cathryn G. Mangiamele, Richard Heller

**Affiliations:** 1 Department of Medical Engineering, University of South Florida, Tampa, FL 33612, USA; 2 Frank Reidy Research Center for Bioelectrics, Old Dominion University, Norfolk, VA 23508, USA

**Keywords:** MHC class I, PD-1, tumor microenvironment, gene electrotransfer (GET), melanoma

## Abstract

Resistance to checkpoint-blockade treatments is a challenge in the clinic. Both primary and acquired resistance have become major obstacles, greatly limiting the long-lasting effects and wide application of blockade therapy. Many patients with metastatic melanoma eventually require further therapy. The absence of T-cell infiltration to the tumor site is a well-accepted contributor limiting immune checkpoint inhibitor efficacy. In this study, we combined intratumoral injection of plasmid IL-12 with electrotransfer and anti-PD-1 in metastatic B16F10 melanoma tumor model to increase tumor-infiltrating lymphocytes and improve therapeutic efficacy. We showed that effective anti-tumor responses required a subset of tumor-infiltrating CD8^+^ and CD4^+^ T cells. Additionally, the combination therapy induced higher MHC-I surface expression on tumor cells to hamper tumor cells escaping from immune recognition. Furthermore, we found that activating T cells by exposure to IL-12 resulted in tumors sensitized to anti-PD-1 treatment, suggesting a therapeutic strategy to improve responses to checkpoint blockade.

## 1. Introduction

The immune system is a key player in cancer development and progression. The interaction between the tumor and surrounding immune cells is constant and complex, leading either to the inhibition or stimulation of tumor growth. The role of the immune system in the rejection of solid tumors has been well demonstrated in both carcinogen-induced and spontaneous experimental tumor models [1]. The immune-mediated eradication of established tumors depends on several factors, including the presence of tumor antigen-primed CD8^+^ effector T cells, the ability of such T cells to traffic to sites of tumor growth, the persistence of cells in sufficient concentrations at the tumor site, and the capacity of the T cells to maintain cytotoxic effector functions in the face of local immunosuppressive mechanisms. Immunotherapy has revolutionized cancer therapy. The suppression of tumor growth can be achieved via increasing tumor-infiltrating lymphocytes, such as CD8^+^ T cells [2]. A lack of T cells in tumors can lead to resistance to immunotherapy. Thus, strategies aimed at maintaining effector T cells within the tumor microenvironment following exposure to specific cytokines, i.e., IL-12, combined with anti-PD1, are a high priority [3,4].

Immune checkpoint inhibitors, such as antibodies against PD-1, would be a prioritized therapeutic modality when CD8^+^ T cells with a high expression of PD-1 are present in the tumor milieu. This approach has been demonstrated to be effective against a large number of cancer types, including melanoma, non-small-cell lung cancer, and renal cancer [5,6]. In addition, checkpoint inhibitors, such as anti-PD1, have recently been shown to substantially extend survival in melanoma patients [7,8]. Despite significant clinical benefits, a high number of cancer patients relapse within a few months after initiation of therapy [9,10,11,12]. The prognosis of melanoma remains poor, especially when the disease also involves mucosal surfaces [13]. A limited number of tumor-infiltrating immune cells might be one of the mechanisms leading to resistance to PD1 inhibition in patients [14]. Evidence from clinical cases demonstrated that a large proportion of solid cancers appeared immune privileged to lymphocytic infiltrate or were non-immunogenic (immunologically “cold”) and thus protected from cytotoxic attack by T lymphocytes [15]. In patients who resist immune checkpoint blockades, CD8^+^ T cells localize peritumorally, whereas they accumulate in the tumor mass in patients who respond to immune checkpoint blockade, suggesting that strategies aimed at promoting T-cell infiltration into the tumor mass are indispensable for the induction of therapeutic immunity [16,17]. Given that the function of some cytokines is recruiting and cueing the proliferation of lymphocytes to bolster the immune response, combining checkpoint blockade with cytokines might improve response rates [18,19,20]. Prior studies in our lab suggest that the local expression of plasmid-encoding IL-12 (pIL-12) delivered by gene electrotransfer (GET) has the function of increasing immune cell infiltration into the tumor microenvironment [4].

The trigger of an anti-tumor immune response starts with major histocompatibility complex class I (MHC-I) and II (MHC-II) presenting tumor antigens to CD8^+^ and CD4^+^ T cells, respectively. That is, MHC molecules play a pivotal role in the initiation and subsequent anti-tumor immune response [21,22]. However, evidence from clinical cases demonstrates that a large proportion of solid cancers appear to down-regulate or lose MHC-I antigen presentation [21,23,24]. We previously demonstrated that the intra-tumor delivery of plasmid IL-12 with GET resulted in a highly efficient anti-tumor response [25,26,27]. Herein, we expanded upon our previous findings to demonstrate that pIL-12 GET combined with anti-PD1 in metastatic melanoma tumor model could shift poorly immunogenic tumors milieu into highly inflamed immunologically active lesions. One mechanism behind this observation is an increase in MHC-I surface expression on tumor cells, thus, converting tumor cells into antigen-presenting cells combined with IL-12 working to recruit tumor-infiltrating lymphocytes leading to the eradication of established tumors and generating tumor-specific memory.

## 2. Materials and Methods

### 2.1. Cells

B16F10 murine melanoma cells (American Type Culture Collection) were maintained in Dulbecco’s Modified Eagle Medium supplemented with 10% FCS and 100 units/mL of penicillin and 100 µg/mL of streptomycin. B16F10-Red-FLuc cells (PerkinElmer, Waltham, MA, USA) were cultured in RPMI 1640 supplemented with 10% FCS.

In vitro electroporation was performed as follows: B16F10 cells (10^7^/mL) were treated with electric pulses (six 500 V/cm, 5 ms pulses with 1.0 s interval) in a 2 mm cuvette with or without plasmid DNA (20 µg) and allowed to grow for 24 h, 48 h or 72 h. The cells were then assessed by flow cytometry for the expression of H-2Kb and PDL1. B16F10 cells at proper confluency were subsequently treated with recombinant mouse IFN-γ or IL-12 (Biolegend, San Diego, CA, USA) at various concentrations as control. Additional analysis was performed using FlowJo software V10 (Tree Star, Inc., San Carlos, CA, USA).

### 2.2. Animals and Tumor Models

Female C57BL/6 mice (6–8 weeks) were obtained from Jackson Laboratories. 1 × 10^6^ B16F10 cells were injected on the left flank (subcutaneous, (s.c.) to establish a primary tumor. Seven days after s.c. injection (Day 0), mice were injected intraperitoneal (i.p.) with 5 × 10^4^ B16F10-Red-FLuc cells to establish peritoneal metastases.

Metastatic spread within the peritoneal cavity was assessed using IVIS^®^ Lumina III in vivo imaging system (IVIS, PerkinElmer, USA) on day 1 and followed every 5 to 10 days thereafter. The anesthetized animals received 15 mg/kg D-luciferin (200 μL/mouse, PerkinElmer, USA) via intraperitoneal injection. Luciferase intensity in the peritoneal cavity exceeding background levels (4~5 × 10^4^ photons/s) was used for the confirmation of tumor growth.

Established subcutaneous tumors (40–60 mm^3^) were treated on days 0, 4, and 7 via intratumoral injection of plasmid encoding mouse pUCMV3-mIL-12 (pIL-12, 50 μg/50μL, Aldevron, Fargo, ND), with GET (ten 600 V/cm, 5 ms pulses with a non-penetrating caliper electrodes with an adjustable distance between electrodes) after anesthetization. The anti-PD-1 (RMP1-14, BioXCell, Lebanon, NH) was injected i.p. at a dose of 200 μg/100 μL on days 0, 4, 7, and 14. The mice were observed every 3 to 5 days, which included tumor measurements, weight, and health status. Tumor volume was calculated using the formula v = 0.52 × length × width^2^. When rechallenged, the mice were injected with 5 × 10^5^ B16F10 cells in the right flank.

For depletion experiments in vivo, the mice were treated with depletion antibodies at a dose of 200 ug/100 μL (i.p., anti-CD4 (clone GK1.5, BioXCell), anti-CD8 (clone 2.43, BioXCell), anti-IFN-γ (XMG1.2, BioXcell) and anti-NK1.1 (clone PK136, BioXCell)) on day −1, 1, 4, 7, 12, and 17. The cellular depletions of CD8^+^ T cells, CD4^+^ T cells, and NK cells were confirmed by flow cytometry of PBMC. The level of depletion was 98% for both T-cell subsets (Figure S3A–E).

### 2.3. Tissue Preparation

The blood was collected from the lateral tail vein at specified time points in tubes containing ethylenediaminetetraacetic acid (EDTA) anticoagulant in order to obtain the plasma. The plasma was stored at −20 degrees for ELISA, and the cells were analyzed by flow cytometry.

The trimmed tumor tissues were fixed in Zinc Fixative (BD Bioscience, Franklin Lakes, NJ, USA), followed by dehydrated and embedded in paraffin in preparation for immunohistochemistry (IHC).

Single cells were isolated from the spleen using a cell strainer (40 µm) followed by treatment with Ammonium-Chloride-Potassium (ACK, A1049201, Altham, MA, USA) to lyse mouse erythrocytes. For tumor-infiltrating lymphocytes (TILs), the tumor tissues were dissociated into single-cell suspensions using the GentleMACS Dissociator (Miltenyi Biotec, Gaithersburg, MD, USA) according to the manufacturer’s instructions. TILs were enriched with 75% Ficoll-Paque premium (GE Healthcare Bio-Science AB, Chicago, IL, USA).

For tumor homogenization, the tissues were snap-frozen in liquid nitrogen and lyophilized overnight, homogenized with GentleMACS Dissociator.

### 2.4. Immunohistochemistry

Paraffin-embedded sections (5 µm) were used for IHC staining with Tyramide Signal Amplification (PerkinElmer, USA). The following primary antibodies were used: rat anti-mouse CD3 (CD3-12), rabbit anti-mouse CD4 (EPR19514), rat anti-mouse CD8a (4SM15), rat anti-mouse Foxp3 (FJK-16S), rabbit anti-mouse PD1 (EPR20665).

The immune cell counts per square millimeter were averaged across replicates. The cut-off values of low versus high immune cells were defined by the midpoint. The slides were examined with a Keyence BZ-X810 microscope using Camera Software BZ-H4A (Keyence Corporation of America, Raleigh, NC, USA). The density of TILs calculation was as follows:

Density of TIL = cells count number/size of image field of view (mm^2^)

Image field of view (height, width, diagonal) = (CCD sensor size (height, width, diagonal))/(objective magnification x adapter magnification).

### 2.5. Cytolytic Activity

Cytolytic T-cell activity was determined by a cell-based flow cytometry assay. As previously described [4], syngeneic B16F10 target cells were labeled with 1 µM carboxyfluorescein succinimidyl ester (CFSE, Biolegend). The purified splenocytes were the effecter cells. Effecter cells (1 × 10^6^) were cultured with CFSE-labeled target B16F10 cells (5 × 10^4^). Then, 4 h after incubation, propidium iodide (PI, 1 μg/mL, Sigma-Aldrich, MO) or 4′,6-Diamidino-2-phenylindole dihydrochloride (DAPI, 1 mg/mL, Sigma-Aldrich) was added. The cytotoxic activity was measured by flow cytometric analysis comparing CFSE^+^PI^+^ or CFSE^+^DAPI^+^ cells with CFSE^+^PI^−^ or CFSE^+^DAPI^−^cells. The percentage of specific lysis (cell/CFSE^+^ PI^+^) was calculated as follows:

%Specific lysis = 100 × (%sample lysis − %basal lysis)/(1 − %basal lysis)

### 2.6. ELISA

The expression of IL-12 and IFN-γ in tumor tissue and plasma was determined using the corresponding ELISA analysis kits from eBioscience (ThermoFisher, Waltham, MA, USA) and R&D Systems (Minneapolis, MN, USA).

### 2.7. Flow Cytometry

The following monoclonal antibodies (mAbs) were used: anti-CD3 (PE vio770, REA 641), anti-CD4 (vioBlue, REA 604), anti-CD8a (APC-vio770, 53-6.7), anti-CD25 (APC, REA 568), anti-CD127 (FITC, A7R34), anti-PD1 (PE, REA 802), anti-NK1.1 (PE, PK136), anti-CD11b (APC, REA 596), anti-Gr-1 (vioBlue, RB6-8C5), anti-CD45 (PerCP vio 700, REA737), anti-CD44 (FITC, REA 664), anti-CD62L (APC, REA 828). The above antibodies were purchased from Miltenyi (Miltenyi Biotech). Anti-NKp46 (FITC, 29A1.4), anti-H-2Kb (APC, AF6-88.5), and anti-PDL1 (PE, 10F.9G2) were purchased from BioLegend and analyzed using MACSquant analyzer 10 (Miltenyi, Bergisch Gladbach, Germany). FlowJo software V10 (Tree Star, Inc., USA) was performed analysis using.

### 2.8. Statistical Analysis

One-way ANOVA was used to compare results from more than two treatment groups. The statistical significance of differences in survival curves was determined by log-rank survival analysis. All of the quantified data are presented as mean ± standard deviation (SD). Statistical analyses were performed with commercially available software (SPSS 16.0 and GraphPad Prism 5, San Diego, CA, USA), and *p* values < 0.05 were considered statistically significant.

## 3. Results

### 3.1. Tumor Regression by Combination Therapy

**Figure 1:**
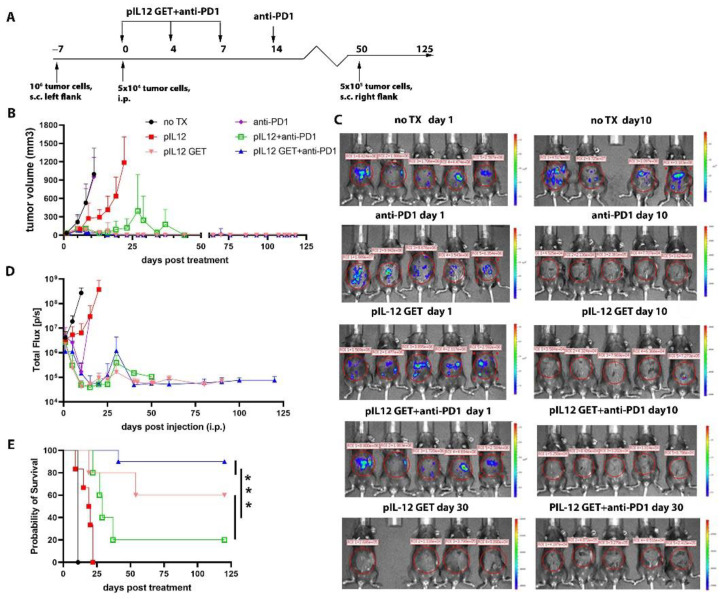
pIL-12 GET in combination with PD1 blockade produces potent rejection of established B16F10 melanoma in a two-tumor model. (**A**) The timeline of combination therapy. (**B**) Data are presented as tumor (s.c.) growth in each group (n = 5/group). (**C**) Representative images of tumors in mice (i.p.) are from in vivo imaging system. (**D**) Quantification of luciferase signal and tumor growth. (**E**) Cumulative survival for each group. ***, *p* < 0.001.

Given the high expression of PD1 on TILs (Supplementary Figure S1A–C), we evaluated whether pIL-12 GET in combination with anti-PD1 could induce a robust anti-tumor immune response. We investigated the anti-tumor activity of pIL-12 GET and the combination of pIL-12 GET with anti-PD1 in the setting of intraperitoneal metastatic B16F10 melanoma (Figure 1A). pIL-12 GET showed impressive attenuation of the primary tumor (s.c.) growth (Figure 1B); however, there was a weak anti-tumor response against the intraperitoneal metastatic tumor (Figure 1C,D). Though the anti-PD1 group demonstrated a response in intraperitoneal metastatic growth, there was not an observed response on the subcutaneous tumor (Figure 1B–D). Compared to pIL-12 GET or anti-PD1 alone, the combination treatment (pIL-12 GET+anti-PD1) improved tumor control, which inhibited both the primary and intraperitoneal tumor progression (Figure 1B). A dramatic reduction in the IVIS signal of intraperitoneal metastases was observed with the combination treatment (Figure 1C–E). Greater than 80–90% of mice treated with the combination achieved long-term survival (Figure 1E). Without GET, the pIL-12 treatment (injection only) showed a weak anti-tumor effect, even when combined with anti-PD1 (Figure 1), which is consistent with previous results [4]. Mice whose primary tumors received pIL12 GET had not only the treated tumor regress but also induced attenuation of tumor growth in distant lesions mediated indirectly by IL-12 priming in treated tumors. Anti-tumor effects seen in distant contralateral lesions are, therefore, likely mediated by changes in tumor immunity generated in the treated tumors. The combination of pIL-12 GET and anti-PD1 immunotherapy further enhanced survival compared to pIL-12 GET alone and led to long-term tumor regression in 90% of treated mice (Figure 1E). Importantly, despite high rates of immune response, pIL-12 GET was associated with minimal systemic toxicity, as mice did not show weight loss (Figure S2A), but some long-term survivors developed regional depigmentation located in the rechallenge site and primary site. (Figure S2B).

### 3.2. Dynamics of Immune Response in Tumor Microenvironment

**Figure 2:**
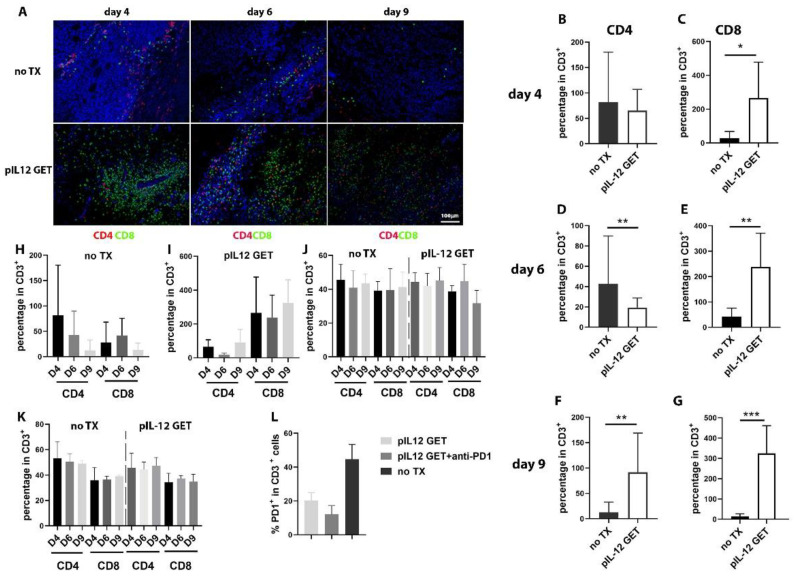
The dynamic changes in regional and system immune response and the expression of PD1 on TILs. (**A**) On day 4, 6, and 9, in a parallel experiment, tumor tissue was dissected and fixed in Zinc fixation buffer, embedded with paraffin, cut and stained. Representative fields (×200) showing CD4 (red) and CD8 (green. Nuclei were stained with DAPI (blue), (n = 5/group/experiment). Representative FACS analysis of TILs. Quantification of CD4^+^ and CD8^+^ in IHC at different timepoints (**B**–**G**). (**H**–**K**) Dynamic changes in regional and system immune response in IHC (**H**,**I**), in blood (**J**) and in splenocytes (**K**). (**L**) CD3^+^PD1^+^ expression in TILs. *, *p* < 0.05, **, *p* < 0.01, ***, *p* < 0.001.

Next, we sought to assess the dynamic change in TILs in tumor-bearing mice. As shown in Figure 2, as the tumors grew in the untreated mice, the number of infiltrating CD8^+^ T cells were observed to be low in the tumor microenvironment (TME) (Figure 2A). pIL-12 GET induced an increase in CD8^+^ TILs (Figure 2A,C,E,G,I). Compared to pIL-12 GET, the absolute number of infiltrating CD4^+^ and CD8^+^ was less, though the relative percentage of CD4 was higher in the no TX (no treatment) group until day 9 (Figure 2F,H). In addition, there was a significantly higher percentage of CD8^+^/PD1^+^ T cells in the pIL-12 GET group compared to the combination group (Figure 2L).

### 3.3. Endogenous IFN-γ Response within Tumors with pIL-12 GET Combination Treatment

**Figure 3:**
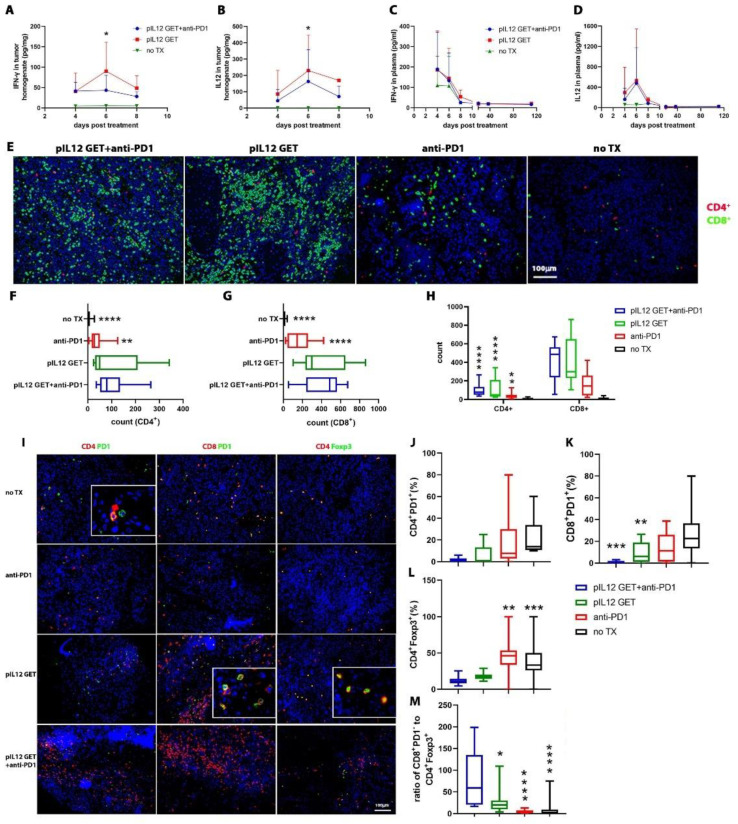
pIL-12 GET combination therapy induces pronounced immune infiltration of tumors with efficacy dependent on adaptive immune cells. The concentration of (**A**) IFN-γ and (**B**) IL-12 in tumor. The kinetics concentration of (**C**) IFN-γ and (**D**) IL-12 in plasma from long-term survivors. On day 9, tumor tissues were dissected and fixed in Zinc fixation buffer, embedded with paraffin, cut and stained. (**E**) Representative fields (×200) showing CD4 (red) and CD8 (green) in different groups. Tumor-infiltrating lymphocytes quantification of (**F**) CD4^+^ and (**G**) CD8^+^ T cells. (**H**) The total numbers of infiltrating CD8^+^ T cells compared with CD4^+^ T cells. Combination of pIL-12 GET and anti-PD1 decreases the percentages of CD8^+^PD1^+^ and CD4^+^Foxp3^+^ in tumor-infiltrating lymphocytes. (**I**) Representative fields (×200) showing CD4 (red), CD8 (red), PD1 (green), and Foxp3 (green). Nuclei were stained with DAPI (blue), (n = 5 mice/group/experiment). Quantification of (**J**) CD4^+^PD1^+^, (**K**) CD8^+^PD1+, and (**L**) CD4^+^Foxp3^+^ and the ratio of (**M**) CD8^+^PD1^−^to CD4^+^Foxp3^+^. Bar, 50μm. Data are from two independent experiments. * *p* < 0.05; ** *p* < 0.01; *** *p* < 0.001, **** *p* < 0.0001.

The TME was evaluated to determine key aspects of pIL-12 GET and combination treatment related to therapeutic outcomes, particularly the abundance of infiltrating T cells in tumor tissue (Figure 2C) and levels of IFN-γ and IL-12 within the TME. IFN-γ and IL-12 were significantly induced following combination therapy, and pIL-12 GET monotreatment when compared to the no TX group (Figure 3A,B). Achieving similar levels of IFN-γ and IL-12 following combination therapy and pIL-12 GET monotreatment was not surprising as each group received the same intratumor treatment; however, these data demonstrates that the addition of anti-PD-1 did not adversely affect the activity of pIL-12 GET. Intratumoral cytokine levels increased on day 4 and remained elevated out to day 8, which was the last day that intratumoral levels could be assessed. As shown in Figure 1B, with treatment, the tumor volume shrank from around 50 mm^3^ to around 10 mm^3^, at which point it was difficult to obtain tumor homogenate.

In consideration of the potential for toxicity due to increased levels of cytokines in the circulation, plasma samples were assayed to determine levels of IFN-γ and IL-12. Compared to the no TX group, there was no significant difference in the pIL-12 GET or combination group. However, at the early time points, there was a slight increase in the level of IFN-γ and IL-12 in plasma in the pIL-12 GET treatment and combination groups (Figure 3C,D). The data indicated that the levels of IFN-γ and IL-12 in the plasma were lower in long-term surviving mice.

### 3.4. Modifications of Immune Cell Infiltrate in Tumor Microenvironment

To understand the cellular mechanisms underlying the observed therapeutic effect of the combination therapy, we sought to further characterize TILs induced by IL-12. The results from Figure 2A and Figure 3E indicated that there was an increased level of TILs and suggested that these T cells could derive from the blood circulation. Although there is a significant difference with respect to CD4^+^ T cells compared to no TX and anti-PD1 group, the overall number of CD4^+^ T cells in tumors was low (Figure 3F). Furthermore, the total number of infiltrating CD8^+^ T cells per tumor size was significantly increased in pIL-12 GET and combination groups (Figure 3G) compared to no TX group. The abundance of antigen-specific CD8^+^ T cells correlated with regional vitiligo in treated animals, which is associated with favorable responses to immunotherapy in clinical studies (Figure S2B). Thus, combination therapy elicited substantial remodeling of the TME with contributions from CD4^+^ and CD8^+^ T cells, particularly the latter.

By assessing exhausted T cells within the tumors with IHC, we found that CD4^+^PD1^+^ and CD8^+^PD1^+^ T cells were infrequent in combination therapy (Figure 3I–K). Notably, pIL-12 GET and combination therapy induced tumor infiltration by effector CD4^+^ and CD8^+^ T cells (Figure 3J,K). Interestingly, we found the frequency of CD4^+^Foxp3^+^ T cells in both pIL-12 GET and combination group to be lower than that of the control groups (Figure 3L) and the ratio of CD8^+^PD1^−^ to CD4^+^Foxp3^+^ to be higher (Figure 3M). The cells expressing the exhaustion marker PD-1 in peripheral blood and spleen were detected with flow cytometry (Figure S3F–I)

The analysis of the IHC results indicated that there were peri-tumoral lymphocytic aggregates with prominent perivascular localization and intra-tumoral lymphocytic penetration in the combination and monotherapy groups as opposed to the no TX group (Figure S4A). Together, these data indicated that T cells infiltrated into the TME in an IL-12-dependent manner, and anti-PD1 immune checkpoint blockade decreased the number of exhausted T cells.

### 3.5. CD8–CD4- and IFN-γ-Dependent Manner of Anti-Tumor Efficacy

**Figure 4:**
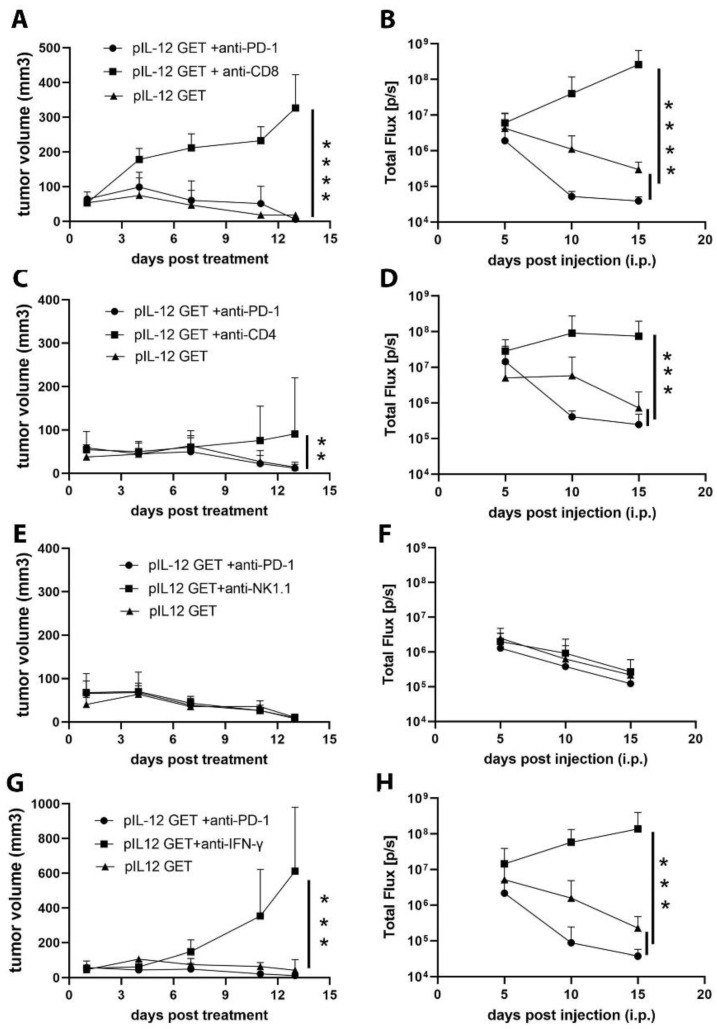
Combination therapy of pIL-12 GET and anti-PD1exerts an anti-tumor effect in a CD8- and CD4-dependent manner. Mice received i.p. injection of (**A**,**B**) anti-CD8, (**C**,**D**) anti-CD4, (**E**,**F**) anti-NK1.1 and (**G**,**H**) anti-IFN-γ on day −1, 0, 2, 4, 7, then followed by twice a week. ** *p* < 0.01; *** *p* < 0.001; **** *p* < 0.0001.

To further delineate the contribution of the host immune response to the anti-tumor effect of combination therapy, we treated tumor-bearing mice with depleting anti-CD4, CD8, NK1.1, or IFN-γ antibodies to eliminate, respectively, CD8^+^ T cells (Figure 4A,B), CD4^+^ T cells (Figure 4C,D), NK cells (Figure 4E,F), and IFN-γ (Figure 4G,H). Although the CD4^+^ T cells were dispensable for pIL-12 GET combination therapeutic efficacy, we found that the combination anti-tumor effect predominantly relied on CD8^+^ T cells (Figure 4D,E). Interestingly, in CD8^+^ T-cell-depleted mice, the therapeutic effects of combination treatment were totally abrogated, including subcutaneous primary tumor and peritoneal metastatic tumor (Figure 4A,B). In IFN-γ-depleted mice, the anti-tumor response decreased, whereas in NK1.1-depleted mice, the combination therapy exerted the same effect on tumor progression, suggesting that NK cells act as weak effectors against tumor progression in this experiment.

### 3.6. Augmentation of Killing Capability in T Cells

**Figure 5:**
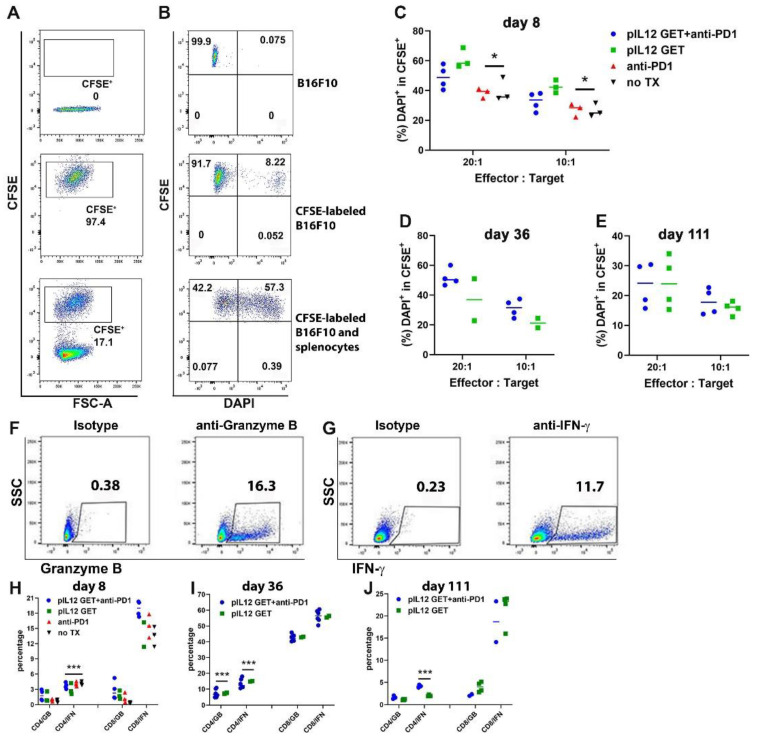
Combination therapy elicits T cells responses and is maintained for a long-term duration. On day 8, 36, 56, and 111, spleens were isolated from the mice (n = 5/group/experiment). After preparing single cells, splenocytes were cocultured at different ratios with CFSE-labeled B16F10 cell targets for 6 h. The cytotoxic activity of T cells was analyzed with FACS. (**A**,**B**) Representative FACS analysis of cytotoxicity of T cells. (**C**–**E**) Quantification of DAPI+/CFSE+ of the target population in comparison to the control population (CFSE-labeled B16F10 cells group). (**F**,**G**) Representative dot plots showing the gating strategy for granzyme B and IFN-γ. (**H**–**J**) Kinetics of IFN-γ or granzyme B production by CD8 and CD4 cells during days 8–111 after the onset of treatment. The experiments were performed three times, yielding similar results. * *p* < 0.05, *** *p* < 0.001.

Given that the principal aim of this study was to use the pIL-12 GET combination with anti-PD1 for generating robust functional T-cells (CD8^+^ PD1^−^), we tested the capacity of the expanded cytotoxic T-cells to kill targeted cancer cells. Using CFSE-stained B16F10 at a 10:1 or 20:1 effector-target ratio, there was around 1.5-fold augmentation in the cytotoxic activity of the splenocytes isolated from pIL-12 GET or combination treatment mice as compared with splenocytes isolated from no TX mice (Figure 5). There was approximately 20% killing in the no TX group, these effector splenocytes were from B16F10 tumor-bearing mice; that is, these effector cells were all primed by B16F10 antigen. Thus, these splenocytes showed a certain cytotoxicity when encountering the same antigen (Figure 4C). We noted that, compared to the no TX group, as shown in Figure 5C,D, the duration of the cytotoxic capacity of CD8^+^T cells elicited following pIL12 GET and combination treatment was prolonged.

To further characterize the cytotoxicity of dominant cells, we sorted CD4^+^ and CD8^+^ T cells from the spleen of tumor-free mice on day 56 and examined the cytotoxic capacity of these two cell types with flow cytometry. Compared to sorted CD4^+^ T cells, sorted CD8^+^ T cells displayed a higher effect on target B16F10 cells (Figure S4B).

To better understand the immunomodulatory effects of pIL-12 GET and the associated mechanism of action in metastatic B16F10, we quantified the key killing mediators granzyme B and IFN-γ. As shown in Figure 5H–J, the CD8^+^ T-cells displayed a higher level of IFN-γ and granzyme B compared with CD4^+^ T cells. Surprisingly, there was no apparent difference in the level of expression of granzyme B on days 8 and 111 (Figure 5H,J). Notably, on day 36, the highest production of IFN-γ and granzyme B was consistent with the more robust cytotoxic ability of the target cells (Figure 5D,I). Splenic CD8^+^ T cells harvested from either no TX group or pIL-12 GET treatment group, these cells were capable of producing the key effector cytokines IFN-γ and granzyme B (Figure 5H–J), the splenic CD8^+^ T cells from tumor-bearing mice induced higher IFN-γ production from day 8 compared to CD4^+^ T cells (Figure 5H). The production of IFN-γ from CD8^+^ T cells was up to the peak on day 36 (Figure 5I) and followed by a decrease on day 111 in the pIL-12 GET and combination treatment group (Figure 5J). However, the tumor was eliminated after day 36. Interestingly, the granzyme B production from CD8^+^ T cells was higher than from CD4^+^ T cells on day 36. These results demonstrate that T-cell behavior in vitro might not accurately predict their activation and function in the tumor context.

### 3.7. Mediating Long-Term Tumor Protection

**Figure 6:**
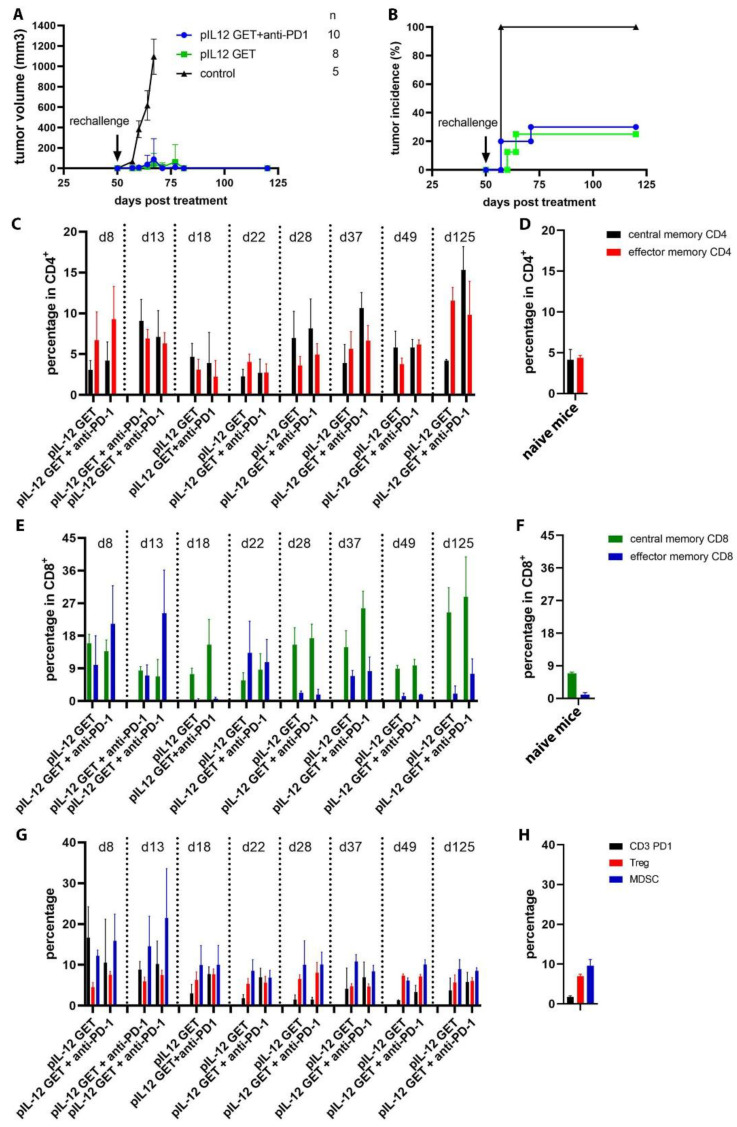
Combination therapy results in complete regression of established tumors and induces protective memory in multi-tumor models. (**A**,**B**) The tumor-free mice rejected a rechallenge with B16F10 tumor cells. (**C**,**E**,**G**) Dynamic changes of immune cells in peripheral blood circulation from the long-term survival mice. (**D**,**F**,**H**) Control data were from naïve mice peripheral blood.

To determine whether pIL-12 GET and combination therapy could elicit durable tumor-specific T-cell responses, we rechallenged the long-term surviving mice (>50 days). Using naive mice as controls, all of the surviving mice were rechallenged with injections of 5 × 10^5^ B16F10 tumor cells in the right flank on Day 50. One hundred percent of the naive mice developed tumors 7 days after the rechallenge. Compared to the naïve mice, a majority of the surviving mice exhibited complete protection (Figure 6A,B). This survival pattern indicated that the surviving mice have long-term immunologic memory against B16F10 cells, and this protection depended on memory T cells. The central memory (CD44^+^ CD62L^+^) and effector memory (CD44^+^ CD62L^−^) was identified by flow cytometry (Figure 6C–F). Nonetheless, these results showed that a predominant amount of protective immunity afforded by pIL-12 GET and combination with anti-PD1 is driven by CD4^+^ and CD8^+^ T cells, and this protection can be enhanced by antigen boost, at which the increased percentage of the effector memory and central memory CD4^+^ and CD8^+^ T cells after rechallenge (Figure 6C,E; 50 days post rechallenge). To further explore the immune system balance, we detected CD3^+^ PD1^+^ T cells, Treg (CD3^+^, CD4^+^, CD25^+^, and CD127^−^), and myeloid-derived suppressor cells (MDSCs, CD11b^+^ Gr-1^+^) as well (Figure 6G).

### 3.8. pIL-12 GET Derived-Tumor Immune Modulatory Program

**Figure 7:**
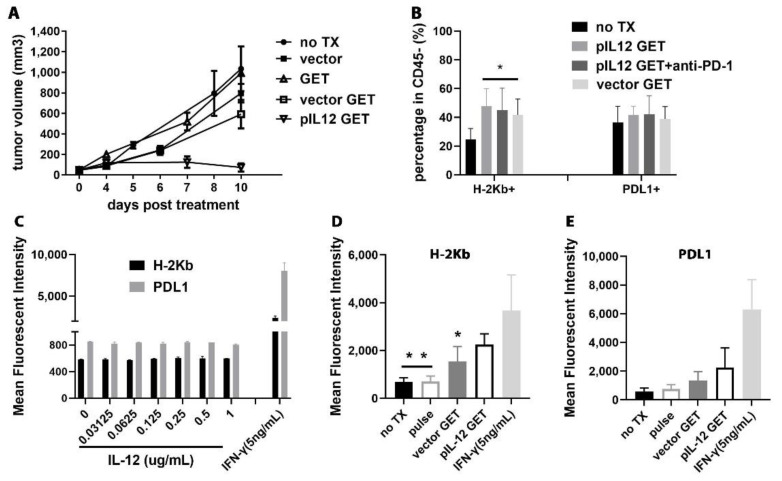
Effect of pIL-12 GET and IFN-γ on H-2Kb and PDL1 expression in B16F10 melanoma. (**A**) Data are presented as tumor (s.c.) growth in each group (n = 5/group). (**B**) Tumor-bearing mice were treated at day 0 with pIL12 GET and anti-PD1. 24 h after treatment, the tumor tissues were isolated, and the single cells were prepared for flow cytometry testing H-2Kb and PDL1 surface expression on B16F10. (**C**). B16F10 cells were seeded into 6-well plate after treatment with electric pulses in the presence of recombination of mouse IL-12 (IFN-γ group as positive control) and grown for 24 h. After that, the expression of H-2Kb and PDL1 were assessed by flow cytometry (**D**,**E**). Flow cytometry data of H-2Kb and PDL1 surface expression on B16F10 cells in different treatments. After treating with electric pulses, B16F10 cells were seeded into 6-well plate for 24 h culture in 6-well plate. * *p* < 0.05, ** *p* < 0.01.

A slight growth delay in the vector GET group was observed in the empty vector-treated mice as compared with the untreated mice (Figure 7A). With respect to immune escape strategies aimed to avoid T-cell recognition, including the loss of tumor MHC class I expression, which has proven to have a negative effect on the clinical outcome of cancer immunotherapy, including treatment with antibodies blocking immune checkpoint molecules, we detected the major histocompatibility complex class I (MHC-I) surface expression on tumor cells from melanoma tumor tissue (H-2Kb). We show that MHC-I was upregulated in B16F10 tumor-bearing mice treated with vector GET and pIL-12 GET.

One role of IL-12 is to recruit and activate NK cells and T cells, which then release IFN-γ [28,29,30]. Therefore, we next sought to determine whether IFN-γ exhibited immune-modulatory functions in the context of B16F10 tumors, such as the decreased expression of a negative immune regulatory factor or the increased expression of positive immune markers. The B16F10 cells were co-cultured with various doses of IFN-γ for 24, 48, and 72 h in culture, followed by flow cytometry analysis. This resulted in dramatically higher expression of H-2Kb and PDL1 in a dose-dependent response but was time-independent (Supplementary Figure S4C). Compared to PDL1 expression, H-2Kb is more susceptible to IFN-γ regulation at the same dose (Supplementary Figure S4C).

To confirm that the agents upregulating the expression of MHC-I was not from IL-12 encoded by pIL-12, we incubated the B16F10 cells with different concentrations of mouse IL-12. As shown in Figure 7C, there was a weak effect of IL-12 and pulse only on H-2Kb and PDL1 expression. To further investigate the function of pIL-12 GET on the expression of MHC-I and PDL1, we performed in vitro tests. pIL-12 GET enhanced H-2Kb and PDL1 expression on B16F10 cells (Figure 7D,E). Obviously, there was a synergy between plasmid IL-12 GET and IFN-γ, and there are more complex effectors that influenced the expression of H-2Kb and PDL1 on B16F10 melanoma in TME.

Previous studies have shown that IFN-γ signaling is triggered after engagement to the heterodimeric receptor of IFN-γ (IFN-γR), which is ubiquitously expressed on virtually all normal cell surfaces [31]. We next measured the IFN-γR expression on B16F10 cells with flow cytometry. We found no evidence for inhibitory or stimulatory effects of IL-12, pIL12 GET, and IFN-γ-treated B16F10 cells on IFN-γR expression (data not shown). These data strongly suggested that IFN-γ and plasmid vector pUMVC3 (data not shown), which was used in the current study, increased the expression of H-2Kb and PDL1on B16F10 cells. Thus, the immunomodulatory effects of pIL-12 GET likely involve the upregulation of class-I antigen presentation to potentiate CD8^+^ T cell responses. These strategies may be useful to potentiate anti-tumor immunity and responses to checkpoint inhibition in immune-refractory melanoma cancers.

## 4. Discussion

A successful anti-tumor immune response requires a few key steps: (a) the capture of tumor antigens by MHC molecules, which are expressed on antigen-presenting cells (APCs), (b) the activation and expansion of CD4^+^ and/or CD8^+^ T cells, (c) the production of inflammatory cytokines (i.e., IFN-γ), and cytotoxin (i.e., granzymes B, perforin). The status of the tumor microenvironment has a great deal of influence on the effectiveness of immunotherapy approaches. A cold, altered, or immunosuppressed microenvironment that has low levels of lymphocytic infiltrate or an immunosuppressive environment can negate many immunotherapeutic approaches. This type of environment has led to clinical benefit being elusive for most patients with cancer, resulting in low response rates and a lack of complete responses. The insufficient number of highly functional immune effector cells in the tumor microenvironment (TME) is one of the key elements. Modifying the environment to be hot where there is an increased infiltrate of activated T cells and/or increased expression of checkpoints can make the tumor(s) more responsive to certain immunotherapeutic approaches [32,33,34,35].

The intratumoral administration of plasmid IL-12 provides a critical bridge between innate and adaptive immunity to meet the above requirements by recruiting and activating natural killer (NK), NKT, and CD4^+^ and/or CD8^+^ T cells into the tumor microenvironment with less toxicity. There is thus an unmet clinical need for therapeutic strategies to convert the TME to an effective functionally inflamed immune landscape able to promote and sustain significant clinical benefit [35,36,37,38].

The observations of the activated antigen-directed TILs in the tumor milieu were associated with favorable prognosis in cancer patients [39,40,41,42]. Immune checkpoint inhibitors have been approved based on durable efficacy [7,8]. However, despite these promising long-term clinical responses, the majority of patients fail to respond to immune checkpoint inhibitors, demonstrating primary or acquired resistance [43,44,45,46]. Thus, there is an urgent need to improve its efficacy and reduce resistance.

Electroporation, with high intensity and short duration, has been used in vivo to permeabilize the plasma membrane of cells for the delivery of plasmid DNA and cytotoxic agents. We have previously demonstrated that by modifying the electrotransfer pulse parameters, an expression pattern for the delivered transgene can be obtained that can lead to the desired therapeutic outcome [47]. Due to its safety, efficacy, flexibility, ease of application, and low cost, gene electrotransfer is becoming popular in clinical trials. Currently, there are over 100 clinical trials (ClinicalTrials.gov) using electroporation to deliver plasmid DNA. Therefore, localized pIL12 GET may be best realized in combination with immune checkpoint inhibitors [48,49,50,51,52,53,54].

Based on the previous results, without GET, pIL-12 injection only results in very low levels of expression and induces a weak anti-tumor effect [4,47,49]. One of the major obstacles in non-viral gene delivery is the interstitial transport of large nucleic acids and their carriers (such as plasmid DNA). Genes must first gain access to the cell surface before they are able to enter cells and achieve protein expression. Plasmid DNA can be introduced into cells using electric pulses. In order to obtain controllable expression of IL-12, we have optimized the parameters of electroporation to attenuate tumor growth [55].

Dependent on the goals of the gene transfer approach, gene therapy can be performed to achieve two different delivery results: transient gene expression and stable transfection. The main discrepancy between transient and stable transfection is that during transient transfection, the gene of interest fails to integrate with the host genome and is expressed temporarily within the host for a short term, whereas, in a stable transfection, the gene of interest integrates with the host genome and is sustained long term for several generations. With respect to the main purpose of pIL-12 GET, we performed transient gene expression as we did not want to have a high or long expression of pIL-12, which could lead to toxicity as well as induce immunosuppression instead of immunostimulation. Furthermore, transient gene expression avoids the high risk of carcinogenic mutation after genomic integration.

Gene electrotransfer with IL-12 is an effective anti-tumor treatment already used in preclinical and clinical oncology [30,56,57,58,59]. A key driver in amplifying this local therapy into a systemic response is the magnitude and composition of immune infiltrate in the treated tumor. While intratumoral IL-12 typically increases the density of CD3^+^ T cells, this infiltrate is educated by the tumor milieu into exhausted T cells. To encourage a more favorable on-treatment tumor microenvironment, we explored combining pIL-12 GET with anti-PD-1 to productively engage a diverse subset of lymphocytes. This therapy yielded high ratios of CD8^+^/PD1^−^ cells in the tumor and decreased the numbers of Tregs, creating a tumor microenvironment conducive to the influx of CD8^+^ T cells. The infiltration of highly active CD8^+^/PD1^−^ T cells was associated with tumor remission and significantly extended survival in tumor-bearing mice. More importantly, long-term tumor-free mice were resistant to tumor growth following subsequent tumor rechallenge and were shown to have induction of long-term tumor antigen-specific immunological memory. These observations are critical to establishing a therapeutic approach that can prevent regrowth or treat metastatic disease [54,60,61,62]. Survival studies in our peritoneal metastatic tumor model showed a marked decrease in mortality among mice with combination treatment vs. pIL-12 GET alone or anti-PD1 inhibitor alone. There were overlapping effects of IL12-GET and combination therapy in the primary subcutaneous tumor.

Durable anti-tumor immune protection can be defined as a subset of persistent antigen-specific memory T cells, which could mediate and coordinate a faster, stronger, and more prolonged response to tumor antigen re-encounter. In the current study, the induction of persistent tumor immunity by pIL-12 GET combined with anti-PD1 in tumor-bearing mice was supported by (a) successful resistance to tumor cells rechallenge, (b) improved levels of antigen-specific cytotoxic T lymphocytes responses in vitro, (c) elevation in the total numbers of CD4 and CD8 T cell populations in the tumor milieu, (d) raised number of activated/memory CD8^+^ T cell subpopulations in the spleens of treated mice. These results suggest that pIL-12 GET combined with anti-PD1 worked to maintain memory and/or activated T cells, initiated and potentially modulated host anti-tumor immune responses in mouse models with primary subcutaneous tumor and peritoneal metastases simultaneously. Subsequently, the release by electric pulse-induced cellular manipulation and re-expressed tumor-associated antigens by host antigen-presenting cells may aid, in part, the generation and maintenance of memory T cells [63,64,65,66]. While the full mechanism of immunologic memory remains unknown, it has been suggested that the successful generation of long-lived memory T cells is dependent on several factors, including the cytokines in the tumor microenvironment [66]. Therefore, we assessed the role of pIL-12 GET combined with an anti-PD1 inhibitor in recruiting and activating the lymphocytes, which may play a crucial role in mediating tumor regression and establishing long-term immunity.

Immune-escape strategies of tumor cells aimed to avoid T-cell recognition, including the loss of tumor MHC class I expression, are commonly found in malignant cells [21,24]. Studies have shown that the upregulated expression of MHC class I molecules, led to more efficient processing and presentation of MHC class I-associated peptides at the cell surface in a variety of cells including different types of tumors [67,68]. It is clear that IL-12 induced cell-derived IFN-γ therapy and local secretion of IFN-γ at the tumor site plays an important role in increasing MHC class I expression. However, in the current context, pIL-12 GET converted the tumor cells into “antigen-presenting cells”, which heightened the opportunity of T cell activation. Interestingly, the data from in vitro studies indicated that plasmid vector pUMVC3 has the capability of increasing the MHC class I surface expression on tumor cells (data not shown) as well. The mechanisms of plasmid vector pUMVC3 upregulation of MHC class I surface expression on tumor cells requires further study to fully understand.

In addition, the increased PDL1 expression on tumor cells is also important to monitor (Figure 7). Evidence from clinical cases indicated that response rates to immune checkpoint blockade range from 36% to 100% for PDL1-positive tumors, while patients whose tumors do not express PDL1 can experience a response rate ranging from 0% to 17% [69,70,71]. The results from our lab found that PDL1 is an inducible and highly dynamic ligand that can change over time (data not shown). Importantly, IFN-γ can induce the production of PDL1 in cancer cells [72,73,74,75]. In the study reported here, the induction of PDL1 by IFN-γ did not overwhelm the anti-tumor effects of IFN-γ, which is partly consistent with previous findings [76]. Notably, we also found that plasmid vector pUMVC3 could increase the PDL1 surface expression on B16F10 tumor cells. Given that the efficacy of immune checkpoint inhibitors depends on the activated T cells in the tumor milieu, which is modulated by chemokine/chemokine receptor interaction [77,78], this prompted us to evaluate chemokine networks.

In summary, we showed that pIL-12 GET alters the tumor microenvironment from the suppressive condition to an anti-tumor milieu. These data highlight CD44^+^ T memory cells in circulation as more representative of cells at immune sites and underscore the importance of evaluating the peripheral blood when making determinations about immune surveillance being able to successfully prevent tumor relapse and metastasis. More broadly speaking, these findings may guide the development of combination cancer therapies to make tumors more accessible for targeted immune therapy and vaccination to establish long-term anti-tumor immunity in patients.

## Data Availability

All the data associated with this study are present in the paper or the Supplementary Materials. Materials are available upon request from the corresponding author.

## Supplementary Materials

The following supporting information can be downloaded at: https://www.mdpi.com/article/10.3390/pharmaceutics14112429/s1, Figure S1: Expression of PD1 on TILs; Figure S2: Mouse weight and Regional depigmentation; Figure S3: Efficacy of depletion antibody on day 8 post-treatment. Representative FACS analysis of depletion in peripheral blood, CD4^+^ and CD8^+^ control.; Figure S4: Perivascular localization of lymphocytes; Sorting splenocytes cytotoxicity and IFN-γ titration.
