# Supplementary material for: Modification of the Tumor Microenvironment Enhances Anti-PD-1 Immunotherapy in Metastatic Melanoma
Source: Pharmaceutics. 2022 Nov 10;14(11):2429. doi: 10.3390/pharmaceutics14112429 (PMC9695203; doi:10.3390/pharmaceutics14112429)
Supplement: Supplementary file 1 [file pharmaceutics-14-02429-s001.zip › pharmaceutics-1972010-supplementary.pdf]

Supplementary Material

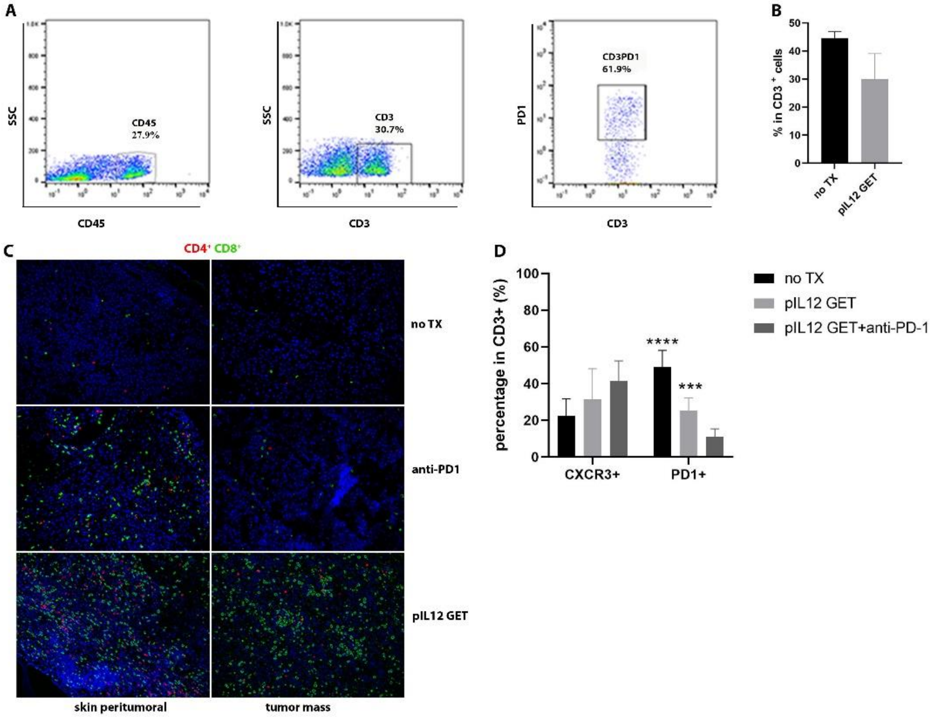


**Supplementary Figure S1. Expression of PD1 on TILs.** (A) Representative FACS analysis of TILs. (B) CD3+PD1+ percentage in TILs. (C) Representative fields (x200) showing T cells infiltrating in skin peritumoral and tumor mass. (D) Tumor-bearing mice were treated at day 0 with pIL12 GET and anti-PD1. 24hrs after treatment, the TILs were prepared for flow cytometry test. CXCR3 and PD1 expression on CD3+ cells (n=5/group/experiment). *** $p < 0.001$; **** $p < 0.0001$.

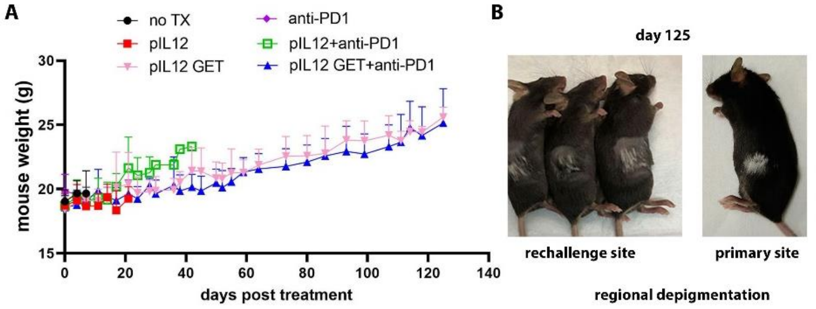


**Supplementary Figure S2. Mouse weight and Regional depigmentation.** (A) Mouse weight. (B) Regional depigmentation located in rechallenge site and primary site.

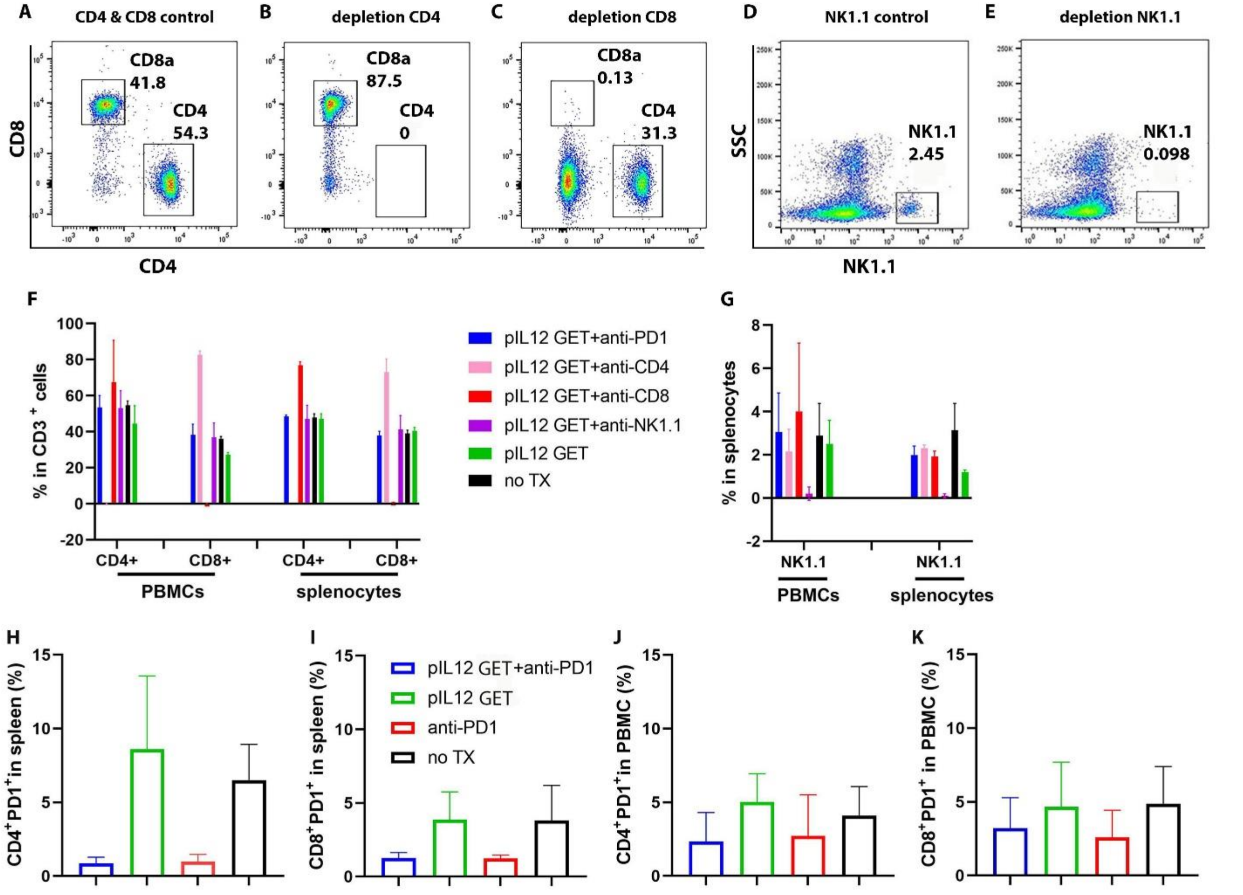


**Supplementary Figure S3. Efficacy of depletion antibody on day 8 post treatment. Representative FACS analysis of depletion in peripheral blood, CD4$^{+}$ and CD8$^{+}$ control**. (A), depletion of CD4$^{+}$ (B), depletion of CD8$^{+}$ (C), NK1.1 control (D), depletion of NK1.1 (E). Quantification of Efficacy of depletion antibody, CD4$^{+}$ and CD8$^{+}$ (F), NK1.1, (G) PD1 expression in CD4$^{+}$ and C8$^{+}$ in peripheral blood and splenocytes (H-K).

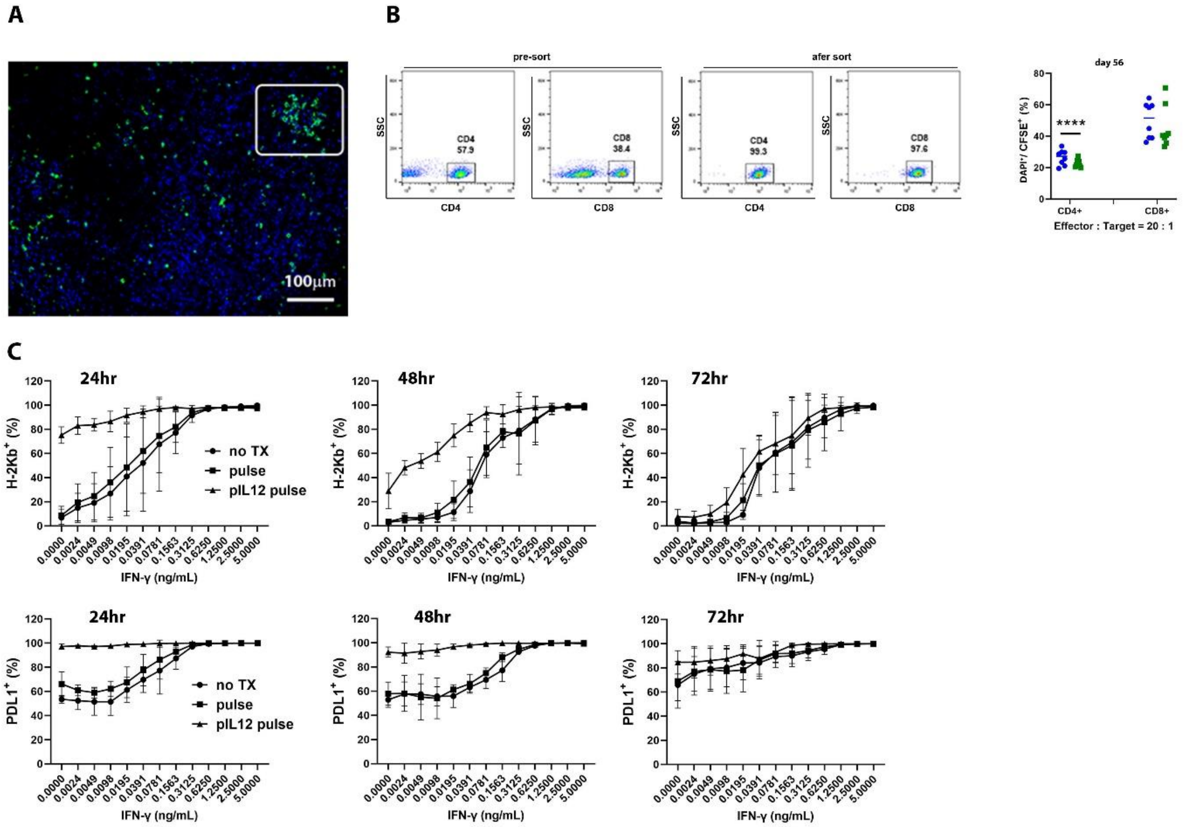


**Supplementary Figure S4. Perivascular localization of lymphocytes; Sorting splenocytes cytotoxicity and IFN-γ titration.** (A) perivascular localization of lymphocytes shown in white square. (B). Representative dot plots showing pre-sort and after sort of CD4+ and CD8+ T cells from splenocytes. (H) Sorted-CD4+, CD8+ T cells as effectors inoculated with CFSE-labeled B16F10 target cells for 6 hours, and then cytotoxic activity was detected with FACS. (C) Different concentration of B16F10 cells, which were treated with electric pulses in the presence of plasmid IL-12, were seeded into 6-well plate with different concentrations of IFN-γ and grown for 24hrs, 48hrs or 72hrs. H-2Kb and PDL1 were assessed by flow cytometry. **** $p < 0.0001$.
